# Cumulative Lead Exposure and Age at Menopause in the Nurses’ Health Study Cohort

**DOI:** 10.1289/ehp.1206399

**Published:** 2014-01-07

**Authors:** Ki-Do Eum, Marc G. Weisskopf, Linda H. Nie, Howard Hu, Susan A. Korrick

**Affiliations:** 1 Department of Environmental Health, and; 2 Department of Epidemiology, Harvard School of Public Health, Boston, Massachusetts, USA; 3 School of Health Sciences, Purdue University, West Lafayette, Indiana, USA; 4 Department of Environmental Health Sciences, University of Michigan School of Public Health, Ann Arbor, Michigan, USA; 5 Channing Division of Network Medicine, Department of Medicine, Brigham and Women’s Hospital and Harvard Medical School, Boston, Massachusetts, USA; * These authors share joint senior authorship.

## Abstract

Background: Early menopause has been associated with many adverse health outcomes, including increased risk of cardiovascular disease morbidity and mortality. Lead has been found to be adversely associated with female reproductive function, but whether exposures experienced by the general population are associated with altered age at menopause has not been explored.

Objective: Our goal was to assess the association between cumulative lead exposure and age at natural menopause.

Methods: Self-reported menopausal status and bone lead concentration measured with K-shell X-ray fluorescence—a biomarker of cumulative lead exposure—were obtained from 434 women participants in the Nurses’ Health Study.

Results: The mean (± SD) age at natural menopause was 50.8 ± 3.6 years. Higher tibia lead level was associated with younger age at menopause. In adjusted analyses, the average age of menopause for women in the highest tertile of tibia lead was 1.21 years younger (95% CI: –2.08, –0.35) than for women in the lowest tertile (*p*-trend = 0.006). Although the number of cases was small (*n* = 23), the odds ratio for early menopause (< 45 years of age) was 5.30 (95% CI: 1.42, 19.78) for women in the highest tertile of tibia lead compared with those in the lowest tertile (*p*-trend = 0.006). There was no association between patella or blood lead and age at menopause.

Conclusions: Our results support an association between low-level cumulative lead exposure and an earlier age at menopause. These data suggest that low-level lead exposure may contribute to menopause-related health outcomes in older women through effects on age at menopause.

Citation: Eum KD, Weisskopf MG, Nie LH, Hu H, Korrick SA. 2014. Cumulative lead exposure and age at menopause in the Nurses’ Health Study Cohort. Environ Health Perspect 122:229–234; http://dx.doi.org/10.1289/ehp.1206399

## Introduction

Early menopause has been associated with several adverse health outcomes including loss of bone mineral density (Gallagher 2007) and cardiovascular disease morbidity (Atsma et al. 2006; Cui et al. 2006) and mortality (Ossewaarde et al. 2005; van der Schouw et al. 1996). Indeed, cardiovascular disease is still the most common cause of death among women worldwide (Mathers et al. 2009). Therefore, mitigation of population exposures to risk factors for early age at menopause could yield significant benefits in terms of reducing chronic disease morbidity and mortality in postmenopausal life.

Adverse female reproductive function effects of lead exposure have been reported in both animal/*in vitro* and human epidemiological studies. Although experimental studies have usually used high exposures and/or exposure routes not reflective of human ones, they have found lead-associated disruption of gonadal function and reproductive hormone production with prenatal as well as later life exposures (Nampoothiri and Gupta 2006; Pillai et al. 2010) and potential impairment of hypothalamic–pituitary–gonadal (HPG) signaling (McGivern et al. 1991). Epidemiological studies have found associations between lead exposure and various reproductive end points, including disruption of reproductive hormones among peripubertal girls (Gollenberg et al. 2010), later puberty (Naicker et al. 2010; Selevan et al. 2003), reduced fertility (Chang et al. 2006; Snijder et al. 2012), and menstrual abnormalities and spontaneous abortion in an occupational group (Tang and Zhu 2003). For example, among 8- to 18-year-old girls participating in the third National Health and Nutrition Examination Survey (NHANES), modestly higher blood lead levels (3 μg/dL vs. 1 μg/dL) were associated with later pubertal development (Selevan et al. 2003). Among battery plant and capacitor factory workers, 52 lead-exposed female workers were found to have a higher prevalence of menstrual abnormalities, including polymenorrhea or hypermenorrhea and spontaneous abortion, than 62 controls randomly sampled from plant workers in non-lead-production departments (Tang and Zhu 2003).

With respect to lead and menopause, most research, including an earlier study in the Nurses’ Health Study cohort (Korrick et al. 2002), has focused on the effects of menopause on blood lead levels (Garrido Latorre et al. 2003; Jackson et al. 2010; Nash et al. 2004; Potula and Kaye 2006; Vahter et al. 2004). Release of lead from bone to blood as a consequence of increased bone turnover following menopause has been proposed as a mechanism that may explain cross-sectional associations between menopause and blood lead levels. However, to our knowledge, only two prior studies have attempted to examine the association between lead exposure and age at menopause (Mendola et al. 2013; Popovic et al. 2005). One was a small study among former smelter workers who were found to have earlier menopause compared with community-based controls, but selection bias or uncontrolled confounding by other occupational exposures could have affected the findings. The second study was a cross-sectional analysis of 1,782 women in NHANES among whom increased odds of natural menopause was seen with higher blood lead levels (Mendola et al. 2013). However, given the cross-sectional analysis and roughly 30-day half-life of blood lead, whether lead caused the earlier menopause or earlier menopause caused the higher blood lead is difficult to determine. We are not aware of any studies that have explored the association between a biomarker of cumulative lead exposure and age at menopause at lower-level, nonoccupational exposures typically experienced by women.

To explore the association between lead exposure and age at menopause, we measured lead concentration in bone—a biomarker of cumulative lead exposure—among older women participants in the Nurses’ Health Study (NHS).

## Methods

*Study population*. The NHS is an ongoing prospective cohort study initiated in 1976 when 121,700 female registered nurses, 30 to 55 years of age and living in 11 U.S. states, completed a questionnaire on their medical history and health-related behaviors (Colditz et al. 1997). The study was designed to assess the relation of diet, lifestyle, and other factors with women’s risk of a wide range of chronic diseases. Since its inception, participants have completed mailed questionnaires every 2 years with response rates of approximately 90%.

The NHS participants in our analyses consisted of a subgroup living in the greater Boston area and assessed in two sequential studies of lead exposure and chronic disease risk in women. In both studies, lead in blood as well as in tibia and patella bone was measured. The first NHS subgroup consisted of 301 women participating in a nested case–control study of lead exposure and hypertension (Korrick et al. 1999). For that study, we invited women to take part if they lived in the greater Boston, Massachusetts, metropolitan area; did not have a history of a major, chronic disease; and were not obese [body mass index (BMI) ≥ 29 kg/m^2^]. Women who had no history of major, chronic disease (no reported diagnosis of hypertension, cardiovascular disease, renal disease, diabetes, or malignancies) were invited to participate as controls from 1990 through 1994, and women who first reported a diagnosis of hypertension between 1990 and 1994 were invited to participate as cases. Controls were frequency matched to cases by 5-year age groups. In total, between 1993 and 1995, 301 NHS participants (101 hypertension cases and 200 controls) agreed to participate and underwent study evaluation, including measurement of their lead levels.

The women in the second Boston-area NHS subgroup were originally recruited for a cohort study of lead exposure and bone density (Weuve et al. 2009). Similar eligibility criteria used for controls in the hypertension study applied here, with participants having no history of chronic diseases (no reported diagnosis of hypertension, cardiovascular disease, renal disease, diabetes, or malignancies) invited to participate from 2001 through 2004. In total, 320 NHS participants completed the bone density study evaluations that included lead measurements. The two substudies were nonoverlapping, with a combined total of 621 unique participants.

We used lead exposure measures, questionnaire, and health information collected in these two Boston area substudies and in the biennial main NHS questionnaires for the current analysis.

*Age at menopause*. Menopausal status was determined on the first NHS questionnaire in 1976 and then again on each biennial questionnaire by asking whether the participants’ menstrual periods had ceased permanently and, if so, at what age and for what reason (natural or surgical). Of the 621 women with lead measurements, 610 had data on age at menopause. Of those women, 449 reported natural menopause, 154 surgical menopause, and seven were missing data on menopause type. Among the 449 with natural menopause, we excluded 15 with missing covariate data, leaving 434 for the current analysis. Thirty-three women reported menopause having occurred between 1957 and 1976, before the first NHS questionnaire. The remaining 401 women underwent menopause between 1976 and 2003. We defined early menopause as natural menopause occurring before 45 years of age (Gallagher 2007).

*Lead exposure assessment*. Participants visited the outpatient General Clinical Research Center (GCRC) of the Brigham and Women’s Hospital for measurement of lead content in their bone by K-shell X-ray fluorescence (KXRF), a noninvasive technique for measuring skeletal lead content that can measure very low lead concentrations (Aro et al. 2000; Nie et al. 2008). The KXRF instrument provides an estimate of bone lead levels normalized to bone mineral content (expressed as micrograms of lead per gram of bone mineral). Negative estimates of bone lead concentrations may occur for lead values close to zero. In epidemiologic studies, use of all point estimates, including negative values, has less bias and greater analytic efficiency than imposing a minimum detectable limit (MDL) and recoding data below the MDL (Kim et al. 1995).

Bone lead measurements were made at each woman’s mid-tibial shaft and patella. These sites are targets for bone lead research because the tibia consists mainly of cortical bone, and the patella of trabecular bone. The estimated half-lives of lead in cortical and trabecular bone in a cohort of older men were on the order of decades and several years, respectively (Wilker et al. 2011). However, a faster rate of decrease in bone mineral density with older age among women compared with men, primarily related to postmenopausal changes in bone physiology (Riggs et al. 1982), likely makes these half-lives shorter in women.

When we began measuring the women’s bone lead, we used an instrument developed by ABIOMED (Danvers, MA). A technical description and validity specifications of this instrument have been published elsewhere (Aro et al. 2000). In 1999, we replaced our prototype ABIOMED instrument with an upgraded instrument designed to be more precise, through changes in the cadmium radiation source, adjustments to the geometry of the measurement procedure, and upgrades in both the system’s software and specific hardware components (Aro et al. 1994). Intercalibration data from persons who were measured on both instruments demonstrated a linear relationship between the two measurements with a slope of 0.87. Using this correction factor, we are able to combine data from our prototype and upgraded KXRF machines (Nie et al. 2008). To reduce the impact of any additional scaling differences in these readings on our epidemiologic analyses, we included a term for lead substudy in our regression models, which effectively adjusts for instrument, because women from the hypertension substudy were assessed on the ABIOMED instrument (Korrick et al. 1999), and women from the bone density substudy were assessed on the upgraded instrument (Weuve et al. 2009).

Whole blood samples were collected in trace-metal-free tubes (with EDTA), and lead levels were analyzed using graphite furnace atomic absorption with Zeeman background-correction (ESA Laboratories, Chelmsford, MA). After every 20 samples, the instrument was calibrated with National Institute of Standards and Technology (NIST) Standard Reference Material (SRM) 955a, lead in blood (NIST, Gaithersburg, MD). To test internal reliability, 10% of samples were run in duplicate; at least 10% of the samples were controls and 10% were blanks. To test external validity, reference samples from the U.S. Centers for Disease Control and Prevention (Atlanta, GA) were measured. Coefficients of variation ranged from 8% for lead concentrations of 10–30 μg/dL to 1% for higher concentrations. The limit of detection (LOD) was 1 μg/dL; values below the LOD were assigned a value of 0.71 μg/dL (1 μg/dL divided by the square root of 2).

*Statistical analysis*. We used ordinary least-squares linear regression to analyze age at menopause as a continuous dependent variable. We used logistic regression to estimate odds ratios (ORs) and 95% CIs for early menopause. We conducted analyses for blood, patella and tibia bone lead biomarkers (separately) categorized into tertiles for models for age at menopause as a continuous variable and early menopause. For trend analyses, we fit models using a single continuous lead biomarker term created by assigning to each woman the median value of her lead biomarker tertile, which reduces the influence of extreme values. In addition, we also report results of trend analyses based on categorizing lead in quintiles. Analyses were adjusted for age at menarche (years), year of birth, substudy group, age at bone lead measurement (years), age at bone lead measurement squared, months of oral contraceptive use, parity (0, 1–2, 3, ≥ 4), and pack-years of smoking assessed at the time of menopause. In sensitivity analyses, we further adjusted for alcohol consumption (< 1, 1–5, 5–10, ≥ 10 g/day) and BMI (< 20, 20–25, ≥ 25) at the time of menopause because these are not consistently associated with menopause. Because age at menopause may affect the use of postmenopausal hormone replacement therapy (HRT), we did not adjust for HRT in our primary analyses. However, we did secondary sensitivity analyses adjusted for HRT use (never, past, current, or premenopausal at the time of bone lead measurement). In addition, to limit the possibility that lead released from bone after menopause affected bone lead concentrations differentially with respect to age at menopause, we performed a sensitivity analysis restricted to women whose bone lead was measured > 5 years after menopause. The 5-year cut point was chosen to approximate the time when the most rapid menopausal bone loss has ended (Greendale et al. 2012; Recker 2011). Only 28 women went through menopause after bone lead measurement, too few to run analyses restricted to that group. We used SAS version 9 (SAS Institute Inc., Cary, NC, USA) for all these analyses. We used R version 3.0.2 (R Project for Statistical Computing, Vienna, Austria) to examine the smoothed, adjusted association between tibia lead and age at menopause with a natural spline. We used Akaike’s information criterion to determine the optimal number of knots. This study was approved by the institutional review board of Brigham and Women’s Hospital. All women gave written consent to participate in studies of lead exposure.

## Results

The mean (± SD) age at bone lead measurement was 61.1 ± 5.9 years (59.4 ± 7.1 years in the hypertension substudy and 62.4 ± 4.3 years in the bone density substudy). The mean age at menopause was 50.8 ± 3.6 years. Of the 434 women in our analyses, 28 were premenopausal at bone lead measurement, with a mean of 3.5 ± 1.7 years between their bone lead measurement and menopause. The remaining women were postmenopausal at bone lead measurement, with a mean time between menopause and subsequent lead measurement of 11.3 ± 6.3 years. Overall the median concentrations of tibia, patella, and blood lead were 10 μg/g [interquartile range (IQR), 4–15], 12 μg/g (IQR, 6–18), and 3 μg/dL (IQR, 2–4), respectively. The distributions of bone lead concentrations by participant characteristics are shown in Table 1. As was observed in our previous case–control study of lead and hypertension in the first NHS subgroup, both tibia and patella lead levels were higher with older age, more pack-years of smoking, and alcohol intake (Korrick et al. 2002).

**Table 1:** Lead exposure biomarkers by general characteristics (*n* = 434) (mean ± SD).

Characteristic	*n*^*a*^	Tibia lead (μg/g)	Patella lead (μg/g)^*b*^	Blood lead (μg/dL)^*c*^
Age at bone lead measure (years)
46–54	62	9.3 ± 7.3	12.8 ± 10.0	2.8 ± 2.2
55–59	100	9.3 ± 7.6	10.3 ± 8.9	2.8 ± 1.7
60–64	141	8.6 ± 9.8	12.0 ± 11.1	3.1 ± 1.9
65–69	101	12.2 ± 10.7	11.7 ± 13.3	3.0 ± 1.5
≥ 70	30	12.9 ± 12.7	16.9 ± 15.0	3.6 ± 2.9
Age at menarche (years)
< 13	188	9.4 ± 9.6	12.2 ± 11.2	3.0 ± 1.9
13	147	9.9 ± 8.9	11.1 ± 11.6	3.0 ± 2.0
> 13	99	11.3 ± 10.5	12.9 ± 11.5	2.9 ± 1.9
Oral contraception use (months)
Never user	221	10.5 ± 10.2	12.3 ± 11.5	3.2 ± 2.0
≤ 24	93	9.8 ± 9.4	12.2 ± 12.5	2.8 ± 1.7
25–60	73	8.7 ± 8.9	10.9 ± 10.7	3.0 ± 2.0
> 60	47	10.1 ± 8.0	11.7 ± 10.0	2.6 ± 1.5
Parity
Nulliparous	22	17.7 ± 14.2	15.7 ± 17.2	2.7 ± 2.2
1	21	12.6 ± 10.6	13.3 ± 10.8	3.4 ± 2.2
2	113	8.3 ± 9.7	10.8 ± 10.8	2.8 ± 1.8
3	135	8.5 ± 8.1	11.5 ± 9.4	3.1 ± 1.9
≥ 4	143	11.2 ± 9.1	12.5 ± 12.6	3.0 ± 1.9
Pack-years of cigarette smoking^*d*^
0	167	9.0 ± 8.6	10.7 ± 10.8	2.7 ± 1.6
1–4	43	8.6 ± 10.8	10.0 ± 8.7	2.7 ± 1.3
5–19	127	10.1 ± 9.9	12.9 ± 11.5	3.2 ± 2.3
20–80	97	12.3 ± 9.9	14.0 ± 13.0	3.5 ± 2.0
Alcohol consumption (g/day)^*d*^
< 1.0	117	8.5 ± 8.4	10.1 ± 9.7	2.6 ± 1.5
1.0–4.9	114	10.2 ± 9.0	11.6 ± 11.8	3.0 ± 1.7
5.0–9.9	67	8.7 ± 10.5	12.1 ± 12.2	2.8 ± 1.8
≥ 10	113	11.5 ± 10.1	13.8 ± 10.7	3.5 ± 2.3
BMI^*d*^
< 20	36	10.4 ± 8.9	13.1 ± 9.6	3.4 ± 1.9
20 to < 25	302	9.9 ± 9.8	12.2 ± 10.8	3.1 ± 2.0
≥ 25	94	10.0 ± 9.4	10.8 ± 13.7	2.5 ± 1.6
HRT use^*e*^
Never	109	11.4 ± 10.4	12.2 ± 13.1	3.9 ± 2.4
Past	152	9.3 ± 10.8	11.4 ± 12.3	3.1 ± 1.6
Current	134	9.9 ± 7.6	12.2 ± 9.2	2.2 ± 1.4
Premenopausal	28	9.1 ± 8.3	13.1 ± 11.5	2.4 ± 1.7
^***a***^Because of missing observations for alcohol consumption (*n* = 22), BMI (*n* = 2), and HRT use (*n* = 11), not all covariates have 434 observations. ^***b***^*n *= 1 missing. ^***c***^*n *= 6 missing.^***d***^At time of menopause. ^***e***^At time of bone lead measurement.				

**Figure 1:**
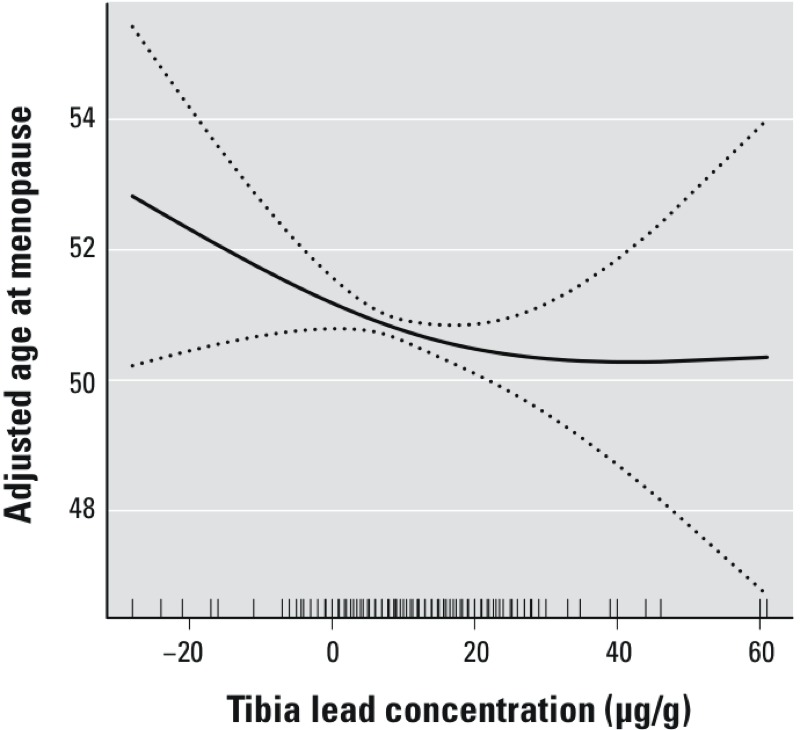
Smoothed (natural spline, 3 knots) association between tibia lead concentration and age at menopause, adjusted for substudy group, age at bone lead measurement, age at bone lead measurement squared, year of birth, age at menarche, months of oral contraceptive use, parity, and pack-years of smoking. The stippled lines indicate the 95% CIs. Short vertical lines on the *x*-axis represent individual women in the study.

Higher tibia lead was associated with a significantly younger age at menopause (Table 2). Compared with women in the lowest tertile of tibia lead, those in the highest tertile were 1.21 years younger at menopause on average (95% CI: –2.08, –0.35; *p*-trend = 0.006). An IQR (11 μg/g) increase in tibia lead concentration was associated with an age 0.89 year younger (95% CI: –1.52, –0.25) at menopause. The analysis of trend using quintiles of tibia lead was also significant (*p* = 0.05). A smooth plot of the adjusted association between tibia lead and age at menopause suggested that the inverse association flattens out somewhat at higher tibia levels (Figure 1), but this is also in the range where there were fewer data. Age at menopause was not associated with patella or blood lead (Table 2).

**Table 2:** Difference^*a*^ (95% CI) in age at natural menopause by lead biomarker concentration (*n* = 434).

Lead biomarker	*n*	Difference in age at natural menopause (years)
Tibia lead tertile (μg/g)
< 6.5	143	Reference
6.5–13	145	–0.80 (–1.67, 0.06)
> 13	146	–1.21 (–2.08, –0.35)
*p* for trend test^*b*^		0.006
Patella lead tertile (μg/g)^*c*^
< 8	134	Reference
8–15	150	–0.32 (–1.18, 0.55)
≥ 15	149	–0.00 (–0.88, 0.87)
*p* for trend test^*b*^		0.99
Blood lead tertile (μg/dL)^*d*^
< 3	192	Reference
3	106	0.08 (–0.80, 0.96)
> 3	130	–0.28 (–1.13, 0.56)
*p* for trend test^*b*^		0.54
^***a***^Adjusted for substudy group, age at bone lead measure, age at bone lead measure squared, year of birth, age at menarche, months of oral contraceptive use, parity, and pack-years of smoking. ^***b***^Calculated using linear regression with a continuous lead biomarker term created by assigning each woman the median value of her lead biomarker tertile. ^***c***^*n *= 1 missing. ^***d***^*n *= 6 missing.		

When age at menopause was dichotomized as early (< 45 years of age) or not, higher tibia lead was associated with early menopause (Table 3). Women in the highest tertile of tibia lead (*n* = 14 cases) had an OR of 5.30 (95% CI: 1.42, 19.78; *p*-trend = 0.006) compared with women in the lowest tertile (*n* = 3 cases). The analysis of trend using quintiles of tibia lead was also significant (*p* = 0.02). For an IQR (11 μg/g) increase in tibia lead concentration, the OR for early menopause was 3.68 (95% CI: 1.46, 9.29). As with analyses of continuous age at menopause, no association was seen for early menopause with blood or patella lead (Table 3).

**Table 3:** OR^*a*^ (95% CI) for early menopause by lead biomarker concentration (*n* = 434).

Lead biomarker	Case/control	Early menopause (< 45 years)
Tibia lead tertile (μg/g)
< 6.5	3/140	Reference
6.5–13	6/139	1.86 (0.44, 7.95)
> 13	14/132	5.30 (1.42, 19.78)
*p* for trend test^*b*^		0.006
Patella lead tertile (μg/g)^*c*^
< 8	7/127	Reference
8–15	11/139	1.24 (0.45, 3.42)
≥ 15	5/144	0.52 (0.15, 1.78)
*p* for trend test^*b*^		0.30
Blood lead tertile (μg/dL)^*d*^
< 3	9/183	Reference
3	7/99	1.43 (0.50, 4.12)
> 3	7/123	1.22 (0.42, 3.58)
*p* for trend test^*b*^		0.68
^***a***^Adjusted for substudy group, age at bone lead measure, age at bone lead measure squared, year of birth, age at menarche, months of oral contraceptive use, parity, and pack-years of smoking. ^***b***^Calculated using linear regression with a continuous lead biomarker term created by assigning each woman the median value of her lead biomarker tertile. ^***c***^*n *= 1 missing. ^***d***^*n *= 6 missing.		

Associations between tibia lead and age at menopause (see Supplemental Material, Table S1) and early menopause (see Supplemental Material, Table S2) were similar to those for the main analysis when we additionally adjusted for BMI and alcohol consumption, or for hormone replacement therapy, or when we restricted the analyses to women who were premenopausal in 1976 (*n* = 401). The association between tibia lead and age at menopause also was similar to the main analysis when we restricted the model to women whose bone lead was measured > 5 years after menopause (see Supplemental Material, Table S1). However, we did not perform this sensitivity analysis for early menopause because of insufficient numbers of cases. The null association of patella lead with menopause was unchanged when restricted to women who were > 5 years after menopause at their bone lead measurement. No sensitivity analyses were performed for the remaining null findings using blood and patella lead measures.

## Discussion

In this study of cumulative lead exposure and age at menopause among women with general environmental exposure to lead, we found a strong association between higher long-term cumulative lead exposure—as measured by lead in the tibia—and younger age at natural menopause. Specifically, women in the highest tertile of tibia lead had five times greater risk of early menopause and experienced menopause > 1 year earlier than women in the lowest tibia lead tertile. From a public health perspective, it is important that these findings were among nonoccupationally exposed women with low lead levels (the average blood lead concentration was 3 μg/dL) comparable to measures in older adult women from the general U.S. population (Campbell and Auinger 2007).

Nonsurgical menopause is triggered by the decline in the number and function of ovarian follicles during the programmed process of ovarian follicle atresia (Broekmans et al. 2009). From at least 300,000 to 400,000 at menarche, the estimated number of primordial follicles falls below 1,000 at the time of menopause, and oocyte quality also diminishes (Faddy et al. 1992). The HPG axis may also contribute to the age-related decline in reproductive function, as a decline in negative feedback from the ovaries alters HPG signaling (Downs and Wise 2009).

Although the mechanism whereby general environmental lead exposure might lead to earlier menopause is uncertain, results of experimental animal models, including studies of nonhuman primates, and *in vitro* studies suggest that lead may affect the female reproductive system in several ways that could contribute to earlier menopause [Doumouchtsis et al. 2009; U.S. Environmental Protection Agency (EPA) 2012]. For example, in an *in vitro* study of human ovarian granulosa cells collected from women undergoing *in vitro* fertilization, cells grown on media that contained lead acetate accumulated lead, which was accompanied by lower levels of p450 aromatase messenger RNA, cytochrome p450 aromatase, and estrogen receptor β proteins than untreated cells (Taupeau et al. 2003). Although the applicability of these *in vitro* findings to the *in vivo* setting is uncertain, aromatase is required for the transformation of androgen to estradiol, and estrogen receptor β mediates estrogen effects in granulosa cells, actions that are essential for follicular growth and maturation, oogenesis, ovulation, and normal luteal functions *in vivo* (Ryan 1982). In addition to direct damage of ovarian cells and ovarian atrophy at high lead levels (Taupeau et al. 2001; Vermande-Van Eck and Meigs 1960), lead also disrupts endocrine function at multiple points along the HPG axis including, for example, altered pituitary gonadotropin production in response to gonadotropin-releasing hormone (Doumouchtsis et al. 2009; U.S. EPA 2012),

Evidence from epidemiologic studies supports the possibility that lead exposures typical of the general population have reproductive effects that could impact menopause. For example, in the National Health And Nutrition Examination Survey (NHANES), lead levels were associated with altered serum follicle-stimulating hormone (FSH) concentrations among premenopausal women (Kreig 2007; Krieg and Feng 2011); however, in another much smaller sample, associations between blood lead and FSH were not seen (Jackson et al. 2011; Pollack et al. 2011). Among 52 occupationally exposed lead battery plant and capacitor factory workers, female lead-exposed workers showed a significantly higher prevalence of polymenorrhea and prolonged and abnormal menstruation than did a control group of 62 women who were randomly sampled workers in administrative or non-lead-production departments (Tang and Zhu 2003). Several epidemiological studies have also found associations between lead exposure and reduced fertility in women, as well as later menarche and pubertal development (U.S. EPA 2012), although the relevance of these end points to menopause is less clear.

Whether lead exposure is associated with age at menopause has been explored in only one occupational study (Popovic et al. 2005) and one cross-sectional study of the general population (Mendola et al. 2013). Among a highly lead exposed group of 108 former smelter employees (Popovic et al. 2005), the mean age at menopause was significantly (*p* = 0.001) younger than among a group of 99 community controls with no known occupational lead exposures. However, the company’s preferential hiring of women for smelter jobs who were unable to have children creates a selection bias—one that likely explains the early age at natural menopause, 43.7 years on average among the lead workers—that limits the validity of these results. Exposures in the second study, a cross-sectional analysis of NHANES data, are applicable to the general population, but the directionality of the observed association of higher blood lead (2–22 μg/dL) with increased odds of natural menopause among 45- to 55-year-old women is uncertain (Mendola et al. 2013). Although this association remained after adjustment for markers of bone turnover or bone density, in a cross-sectional analysis, such measures cannot account for previous postmenopausal releases of lead from bone to blood.

The major strengths of this study include having a large group of nonoccupationally exposed women with bone lead measurements—a cumulative lead exposure marker—and extensive additional covariate data. One of the study’s limitations is that bone lead biomarkers were measured mostly after menopause, thus reverse causation is possible (that is, age at menopause affects lead levels, as opposed to lead affecting age at menopause). Because our bone lead measures were made over a relatively short time interval, women with an earlier age at menopause had more years since menopause at the time of their bone lead measurements than women with later menopause. Although the effect of menopause on bone lead concentration has not been examined empirically, if menopause-related bone loss causes relatively higher bone lead concentration with more time since menopause, this could account for our findings. However, this seems unlikely at face value, but in any case menopause-related bone loss occurs primarily in trabecular (patella) bone rather than cortical (tibia) bone (Riggs et al. 1982). Therefore, reverse causation would be expected to be most apparent for patella lead, but we found associations with tibia lead not patella lead. In addition, the most rapid menopause-related bone loss occurs in the first 5 years after menopause, yet we still saw associations among women who were > 5 years after menopause at the time of bone lead measurement. These findings suggest that possible menopause-associated changes in bone lead are unlikely to explain the observed associations with tibia lead. Nonetheless, only a prospective study with bone lead measured before menopause would answer this question with certainty.

In our study, blood and patella lead likely predominantly reflect postmenopausal lead exposure given their respective half-lives of months to years, and the fact that blood collection and bone lead measurements were done well after most study women were postmenopausal. Thus, a possible explanation for the null blood and patella results is that effects of lead on age at menopause are driven by long-term, premenopausal lead exposures that are reflected better by tibia lead because of its longer half-life (on the order of decades) (Wilker et al. 2011).

In conclusion, this study on the association between bone lead, a measure of long-term lead exposure, and age at menopause suggests that cumulative exposure to lead in a nonoccupationally exposed group is associated with an earlier age at menopause. Given the relation between earlier menopause and many subsequent health problems, these results suggest a pathway by which lead may contribute to the burden of chronic disease in older women. The success in reducing external lead exposures in the United States may mean that women entering menopause today are at less risk of lead-associated earlier age at menopause than we observed, but the possibility remains that further reductions in lead levels could still improve the health of women as they age.

## Supplemental Material

(94 KB) PDF
