# Supplementary material for: Cumulative Lead Exposure and Age at Menopause in the Nurses’ Health Study Cohort
Source: Environ Health Perspect. 2014 Jan 7;122(3):229–34. doi: 10.1289/ehp.1206399 (PMC3948024; doi:10.1289/ehp.1206399)
Supplement: (94 KB) PDF [file ehp.1206399.s001.pdf]

Environmental Health Perspectives doi:10.1289/ehp.1206399

Supplemental Material

Cumulative Lead Exposure and Age at Menopause in the Nurses' Health Study Cohort

Ki-Do Eum, Marc G. Weisskopf, Linda H. Nie, Howard Hu, and Susan A. Korrick

Supplemental Material, Table S1. Difference (95% CI) in age at natural menopause (years) by tibia lead concentration.

Tibia lead tertiles (µg/g)	Difference (95% CI)
Model 1[a]	
< 6.5	Reference
6.5-13	-0.80 (-1.67,0.06)
13+	-1.21 (-2.08, -0.35)
P for trend test	0.006
Model 2[b]	
< 6.5	Reference
6.5-13	-0.74 (-1.62,0.14)
13+	-1.33 (-2.22, -0.44)
P for trend test	0.003
Model 3[c]	
< 6.5	Reference
6.5-13	-0.62 (-1.64,-0.01)
13+	-1.21 (-2.05, -0.37)
P for trend test	0.005
Model 4[d]	
< 6.5	Reference
6.5-13	-0.61 (-1.43,0.21)
13+	-1.05 (-1.88, -0.22)
P for trend test	0.01
Model 5[e]	
< 6.5	Reference
6.5-13	-0.74 (-1.71,0.24)
13+	-0.98 (-1.93, -0.02)
P for trend test	0.05

[a]Default model used for main analysis; adjusted for substudy group, age at bone lead measure, age at bone lead measure squared, year of birth, age at menarche, months of oral contraceptive use, parity, and pack-years of smoking; n = 434. [b]Same as model 1 plus additional adjustment for BMI and alcohol consumption; n = 411. [c]Same as model 1 plus additional adjustment for hormone replacement therapy;n = 423. [d]Same as model 1, but restricted to women with menopause after 1976; n = 401. [e]Same as model 1, but restricted to women who were postmenopausal for more than 5 years before bone lead measurement; n = 321. *P* for trend was calculated using linear regression with a continuous lead biomarker term created by assigning each woman the median value of her lead biomarker tertile.

Supplemental Material, Table S2. Odds ratio (95% CI) for early menopause (< 45years) by tibia lead concentration.

Tibia lead tertiles (µg/g)	OR (95% CI)
Model 1[a]	
< 6.5	Reference
6.5-13	1.86 (0.44,7.95)
13+	5.30 (1.42, 19.78)
P for trend test	0.006
Model 2[b]	
< 6.5	Reference
6.5-13	1.71 (0.39,7.44)
13+	5.36 (1.36, 21.08)
P for trend test	0.008
Model 3[c]	
< 6.5	Reference
6.5-13	1.17 (0.24,5.68)
13+	4.76 (1.25, 18.19)
P for trend test	0.008
Model 4[d]	
< 6.5	Reference
6.5-13	0.77 (0.12,4.98)
13+	6.72 (1.62, 27.84)
P for trend test	0.005

[a]Default model used for main analysis; adjusted for substudy group, age at bone lead measure, age at bone lead measure squared, year of birth, age at menarche, months of oral contraceptive use, parity, and pack-years of smoking; n = 434. [b]Same as model 1 plus additional adjustment for BMI and alcohol consumption; n = 411. [c]Same as model 1 plus additional adjustment for hormone replacement therapy; n = 423. [d]Same as model 1, but restricted to women with menopause after 1976; n = 401. *P* for trend was calculated using linear regression with a continuous lead biomarker term created by assigning each woman the median value of her lead biomarker tertile.
